# Enzymatic Determination of Diglyceride Using an Iridium Nano-Particle Based Single Use, Disposable Biosensor

**DOI:** 10.3390/s100605758

**Published:** 2010-06-08

**Authors:** Shu-Yi Hsu, Brandon Bartling, Christina Wang, Fuh-Sheng Shieu, Chung-Chiun Liu

**Affiliations:** 1 Department of Materials Science and Engineering, National Chung Hsing University, Taichung 40227, Taiwan; E-Mails: g9766119@mail.nchu.edu.tw (S.-Y.H.); fsshieu@dragon.nchu.edu.tw (F.S.S.); 2 Department of Chemical Engineering and Electronics Design Center, Case Western Reserve University, 10900 Euclid Avenue, Cleveland, OH 44106, USA; E-Mail: bxb79@case.edu; 3 Department of Biological Sciences, College of Arts and Sciences, Cornell University, 147 Goldwin Smith Hall, Ithaca, NY 14853-3201, USA; E-Mail: cpw39@cornell.edu

**Keywords:** diglyceride, disposable biosensor, enzyme

## Abstract

A single use, disposable iridium-nano particle contained biosensor had been developed for the determination of diglyceride (DG). In this study hydrogen peroxide, formed through the enzymatic breakdown of DG via lipase, glycerol kinase and glycerol 3-phosphate oxidase, was electrochemically oxidized at an applied potential of +0.5 V *versus* the Ag/AgCl reference electrode. The oxidation current was then used to quantify the diglyceride concentration. Optimum enzyme concentrations and the surfactant loading used were established for successful sensor response. Good linear performance was observed over a DG concentration range of 0 to 25 μM in phosphate buffer and bovine serum media.

## 1. Introduction

Diglyceride (diacylglycerol, DG) is a simple lipid consisting of a glycerol molecule linked through ester bonds to two fatty acids. Its small size and simple composition confer exceptional properties on DG as a lipid intermediate in metabolism, as a component of biological membranes, and as a secondary messenger [1]. Secondary messengers are the essential intermediates linking extracellular stimuli—via receptor activation, to the required intracellular response such as those seen in the nucleus, cytoskeleton or in the case of smooth muscle, the contractile apparatus [2,3]. Early studies identified signaling pathways that proceeded through an apparently linear series of simple steps where an extracellular stimulus via its unique receptor, activated a phospholipase with the resultant hydrolysis of a membrane phospholipid and the production of a secondary messenger [4]. Considerable evidence has accumulated to support secondary messenger functions for DGs as physiological activators of protein kinase C, an enzyme implicated in the regulation of cell activation [5,6], differentiation [7], proliferation [8,9], tumor promotion [10], and other responses [11,12] in a variety of mammalian cell types.

In addition to cell signaling, research has also indicated the health benefits of treatments involving DG. Specifically, research has shown that therapeutic levels of DG can reduce fasting blood triglyceride (triglyceride, TG) levels. The suppression of postprandial TG results in increased blood flow and a reduction in type 2 diabetes. Also observed by researchers was a modification of the level of glycosylated hemoglobin present through DG addition. A change in the glycosylated hemoglobin level helped prevent fat accumulation [13,14], and was shown to extend long-term general health [15]. Researchers have suggested that DG can be used to improve diabetic postprandial lipids [16,17]. Thus, DG has been used in lieu of meals in favor of TG to control diabetes, prevention of arteriosclerosis and other diseases [16,17]. Additional research conducted on dietary effects of DG replacement therapy, as related to diet toxicity and carcinogenicity, in animals [18,19] and humans [20] have shown no negative side effects while still retaining the positive impacts described previously [21,22].

Analytical methods based on chromatographic separation are often used for the determination of mono- and diglycerides in oils, fats, emulsifiers and plant tissues. Extensive research has been carried out using high-performance liquid chromatography (HPLC) [23,24] and HPLC coupled with silver chromatography (Ag–HPLC) [25]. Separation of mono- and diglycerides is also possible by HPLC–gel permeation chromatography [26]. However, a faster measurement technique is desirable for process control and monitoring, for which rapid feedback is needed. The applications of thick film technology to the construction of sensors have been well-documented [27–29]. Compared to other technologies that are available for manufacturing electrodes, such as thin film, thick film biosensors are relatively cost-effective and simple to fabricate and are scalable to mass production. Thick film screen printing involves the use of a paste or ink containing the material of interest such as a metallic catalyst contained within a carbon paste or ink. The ink is pressed onto a flat substrate by a mechanical squeegee through the openings on a stainless steel or polymeric screen transferring a desired pattern onto the substrate. This mass production technology has been used to produce planar patterns of electrodes cost-effectively and with a high degree of reproducibility. In recent years, there is substantial interest in using this technology to produce cost-effective single use, disposable biosensors. However, the quality of the ink is critical, meaning that it must be screen-printing compatible, and has the desired catalytic properties [30,31].

Detection of diglyceride can be carried out using a series of enzymatic reactions, and these reactions in sequence are:

(1) $$\mathrm{Diglyceride}+{2H}_{2}O\overset{\mathrm{lipase}}{\to }\;\mathrm{Glycerol}+\mathrm{Fatty}\;\mathrm{Acid}$$

(2) $$\mathrm{Glycerol}+\mathrm{ATP}\overset{\mathrm{glycerol}\;\mathrm{kinase}}{\to }\;\mathrm{Glycerol}\;\;3-\mathrm{phosphate}+\mathrm{ADP}$$

(3) $$\mathrm{Glycerol}\;\;3-\mathrm{phosphate}+O_{2}\overset{\mathrm{glycerol}\;\;3-\mathrm{phosphate}\;\mathrm{oxidase}}{\to }\;\mathrm{Dihydroxyacetone}\;\mathrm{phosphate}+H_{2}O_{2}$$

Lipase (E.C. 3.1.1.34) is used in the hydrolysis of DG to fatty acid and glycerol. This combined with adenosine 5′-triphosphate disodium salt (ATP), in the presence of glycerol kinase (E.C. 2.7.1.30), is incubated at 37 °C for 1 h. The period of incubation time is needed in order to produce the glycerol 3-phosphate which can then be quantified as shown in Equation (3). The incubation process is well recognized and accepted as necessary in the potential detection of DG. As shown in Equation (3), the glycerol 3-phosphate can be oxidized by glycerol 3-phosphate oxidase (E.C. 1.1.3.21). The H_2_O_2_ produced can then be oxidized electrochemically at a fixed potential, +0.5 V *versus* Ag/AgCl reference electrode. The oxidation current can is used to quantify the original concentration of DG in the test medium.

The above description gives the basic approach and rationale of using thick film screen printing in the development of this single use, disposable biosensor prototype for diglyceride detection. There are three enzymes involved in the reaction sequence. Incubation parameters of the selected enzymatic reactions and the immobilization of the enzyme, glycerol 3-phosphate oxidase, immobilized on the biosensor prototype will be experimentally assessed. Furthermore, the optimal level of each enzyme used will be determined in order to minimize any excess use of the enzyme. We also recognize how test medium pH and the quantity of the surfactant used will affect the performance of this DG biosensor, and these effects will be examined in this study The quantitative measurement of DG present in blood is important. A TG level in blood less than 1.7 mM is considered normal, and a DG level of 50 μM is considered standard. Thus, we will focus on the detection of DG levels from 0 to 50 μM using the single use, disposable biosensor with an iridium nano-particle modified carbon paste electrode in this study.

## 2. Experimental Section

### 2.1. Materials and Reagents

Lipoprotein lipase (E.C. 3.1.1.34) from Pseudomonas sp. (110,000U mg^−1^), glycerol kinase (E.C. 2.7.1.30) from Cellulomonas sp. (GK, 47U mg^−1^), glycerol 3-phosphate oxidase (E.C. 1.1.3.21) from Pediococcus sp. (GPO, 69U mg^−1^), adenosine 5′-triphosphate disodium salt (ATP, □99%), bovine serum albumin (BSA, 98%) and Triton X-100 were purchased from Sigma (St. Louis, MO). Bovine Serum collected from cattle in New Zealand was purchased from Invitrogen (Grand Island, NY). Spectrophotometric readings were conducted using a standard assay kit from Cayman Chemical (Ann Arbor, MI) No.10010303. All other chemical were of analytical grade and used as received.

Phosphate buffer solution (0.1M), were prepared in-house with 0.15M KCl, 0.01M MgCl_2_, KH_2_PO_4_ and K_2_HPO_4_ in appropriate portions, distilled water and adjusted to a pH between 6.5 and 9 using solutions of HCl and NaOH.

### 2.2. Fabrication of Disposable Sensor

**Figure 1.**
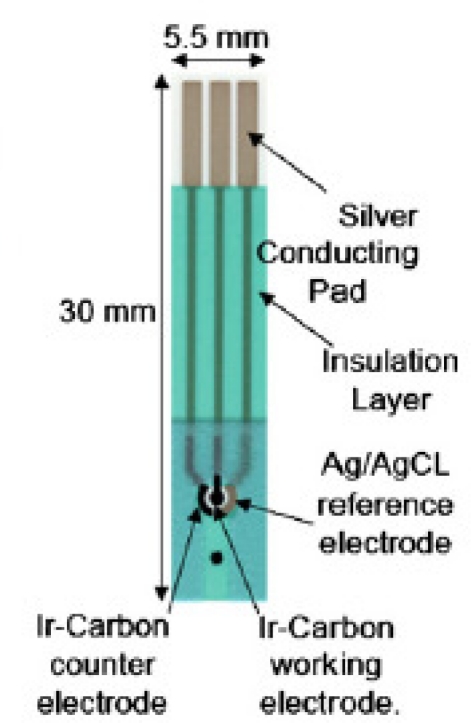
Schematic diagram of the disposable sensor.

This single use, disposable biosensor consisted of three sensing electrode elements: a Ag/AgCl reference electrode (printed using an Ag/AgCl thick film ink), an iridium-containing carbon working electrode, an iridium-containing carbon counter electrode and the sensor was fabricated by the thick-film screen printing using a multi-layer printing approach. The overall dimension of this single-use, disposable DG biosensor was 30 mm × 5.5 mm and the working electrode was 1 mm in diameter. Figure 1 shows the configuration of the single use, disposable biosensor. The physical structure and the preparation of the printable ink for the manufacturing of this basic sensor have been described in details elsewhere [27,29].

### 2.3. Immobilization of Glycerol 3-Phosphate Oxidase on the Iridium-containing Carbon Working Electrode

As mentioned in the enzymatic reactions 1–3, the glycerol 3-phosphate oxidase immobilized on the iridium contained carbon working electrode. A quantity of 0.6 μL 2% glutaraldehyde was first pipetted onto the surface of Ir-carbon working electrode and the sensor was placed at ambient temperature to be air-dried. The glutaraldehyde solution served as the covalent linking agent between the enzyme and the chemical polyethylenimine incorporated in the carbon working electrode. GPO (6U mL^−1^) was then immobilized onto the Ir-carbon working electrode surface. The sensor was stored in a refrigerator at 4 °C for drying overnight. The preparation of the other two enzyme solutions used in this study in described in Section 2.4.

### 2.4. Experimental Procedure

In this study, DG, ATP, lipoprotein lipase (22U mL^−1^), GK (1U mL^−1^) were first added in a 600 μL centrifuge tube with 300 μL of phosphate buffer solution or 1:1 serum-buffer solution and shaken in order to obtain a homogeneous mixture, and then allowed to incubate at 37 °C for 1 h. The 1:1 serum-buffer solution meant that the test medium, the bovine serum was diluted with equal volume of PBS for the detection of DG, the rationale of this dilution is described in a subsequent section. A period of incubation time was needed in order to complete the enzymatic reactions in the DG solution. After incubation, a drop of the testing solution (6 μL) was placed onto the surface of the biosensor using a pipette covering all the three electrodes and amperometric measurements were conducted. Each sensor was used only once during the testing.

All the experiments were conducted at 37 °C. The testing of the sensor was carried out using a CHI 660 C Electrochemical Work Station (CH Instruments, Inc., Austin, TX). Current readings recorded were due to the oxidation of hydrogen peroxide. The current could then be related back to the original concentration of DG in solution. It was recognized that the incubation time for the mixture of DG, ATP, lipoprotein lipase in PBS or 1:1 PBS-serum mixture would be one hour, and the actual detection time of the biosensor would be additional three to ten minutes. While the incubation time was needed for the first portion of the test, it was similar to the incubation time required for any other standard testing methods for DG. The relatively short time needed for detection, ten minutes, of DG was much shorter and simpler than those used in conventional DG detection techniques.

## 3. Results and Discussion

### 3.1. Cyclic-Voltammetric Studies of DG in Phosphate Buffer Solution

**Figure 2.**
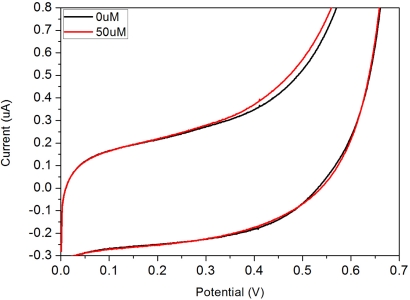
Cyclic-voltammagrams of the disposable biosensor showing the background current and the response current for a 50 μM solution using the disposable biosensor.

Cyclic voltammetric studies were conducted at 37 °C in the absence and the presence of 50 μM DG in a 300 μL test medium. The test medium was 0.1 M PBS solution adjusted to pH 7.4 by adding NaOH. In order to determine an appropriate working potential of this single use, disposable biosensor, an applied potential ranged from 0 to +0.7 V *versus* the printed Ag/AgCl reference electrode was used with a voltage scan rate of 5 mV/s. Figure 2 shows the cyclic voltammagrams obtained using this biosensor prototype.

The electrochemical oxidation of the enzyme generated H_2_O_2_ was observed clearly at +0.5 V (*versus* Ag/AgCl). Therefore, +0.5 V *versus* the printed Ag/AgCl reference electrode was chosen as the sensing potential for the detection of DG using this single use, disposable biosensor. At this selected potential, the current measurements in the presence and absence of DG could be used to quantify the DG in the test medium using the measurements in the development of a single-use disposable DG biosensor. While a potential of +0.5 V, *versus* Ag/AgCl reference electrode, could be a source for concern, related to oxidation of interfering species, it was found in other studies that this was generally not the case [32]. Research performed using the Ir/C sensor technology in the detection of alkaline phosphatase at similar potentials found that levels up to 10 mM L^−1^ of glucose, 5 mg L^−1^ of ascorbic acid and 400 mg L^−1^ of urea showed no impact on the performance of detection. Both concentrations represent physiological levels that well above averages found in an individual.

### 3.2. Reproducibility Studies of the Biosensor Prototype

**Figure 3.**
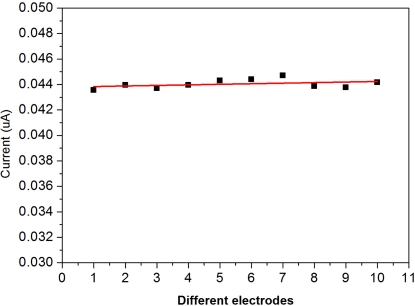
Reproducibility of the disposable biosensor.

The importance of a single use, disposable biosensor includes the high degree of sensitivity and reproducibility of the performance of the biosensor. Furthermore, the biosensor must be able to be produced cost-effectively in order to be a practical single use, disposable prototype. The reproducibility of the biosensor performance was assessed using a randomly selected group of fabricated individual biosensor prototypes. In order to investigate the repeatability of the disposable biosensors, replicate testing was performed. The reproducibility of this disposable biosensor for the detection to a fixed concentration of 5 μM of DG is shown in Figure 3. Testing was conducted by individually applying 6 μL of test solution to each sensor and evaluating the performance at +0.5 V, *versus* Ag/AgCl reference electrode.

The experimental results shown in Figure 3 indicate the high degree of reproducibility of the fabricated single use, disposable biosensors for DG measurements. More than 10 individual single-use, disposable DG biosensor prototypes were used in this study, and the results shown in Figure 3 demonstrates the reproducibility of the biosensor prototypes.

### 3.3. The Effect of pH on the Biosensor Performance

The pH value of the test medium could affect the performance of this biosensor prototype. As described, the enzyme GPO was immobilized on the surface of the iridium nano-particle modified working electrode. The pH effect on the measurement of DG on the GPO immobilized iridium-modified biosensor prototype was assessed experimentally, and a fixed DG concentration of 30 μM was chosen in this study. The test medium was 0.1 M phosphate buffer solution. The pH value in the PBS was adjusting through NaOH or HCl additions to the 1 h incubated samples containing lipase (22U mL^−1^) and glycerol kinase (1U mL^−1^). The pH range studied was 6.5 to 9.0. In each test, the amperometric test time was 10 min and a volume of 6 μL of the test medium was pipetted onto the biosensor covering the surface area of the biosensor prototype.

**Figure 4.**
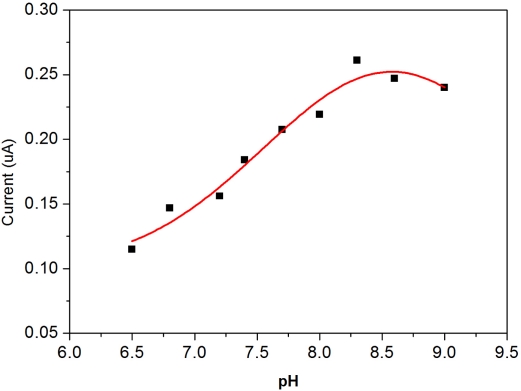
The influence of pH on the response of the disposable biosensor at +0.5 V in the presence of DG (30 μM) in 0.1 M phosphate buffer.

The oxidation current generated by the biosensor prototype based on Equation (2,3) at a fixed DG concentration of 30 μM DG was then measured as a function of pH value in the test medium 0.1 M PBS. Figure 4 shows the effect of the pH value of the test medium of DG detection using this biosensor prototype.

It appears that in PBS, at approximately pH 8.3, the biosensor shows the optimal performance with respect to the pH value of the test medium. Consequently, we chose pH 8.3 as the preferred value for the test medium throughout this study.

### 3.4. Effect of Surfactant on the Performance of the Biosensor Prototype

As mentioned, DG is a glyceride consisting of two fatty acid chains covalently bonded to a glycerol molecule through ester linkages thus making it a non-polar, hydrophobic molecule. The enzymatic reactions [33,34] for detecting the DG may take place at the interface between DG and the aqueous solution. Because of the poor solubility of DG in aqueous solution, the mechanism in which lipase reacts with DG can be regarded as a bi-molecular reaction of enzyme and the relevant surface ligand, which is a heterogeneous, time-consuming reaction [35,36]. Therefore, in order to enhance mixing a surfactant has been suggested to reduce the surface tension of water by adsorbing at the liquid-liquid interface [37]. The surfactant used in this study was Triton X-100 (t-Octylphenoxypolyethoxyethanol). A study on the effect of the surfactant concentration on the DG detection was conducted. Similar to the experimental protocol described previously, the enzyme GPO was immobilized on the working electrode surface.

**Figure 5.**
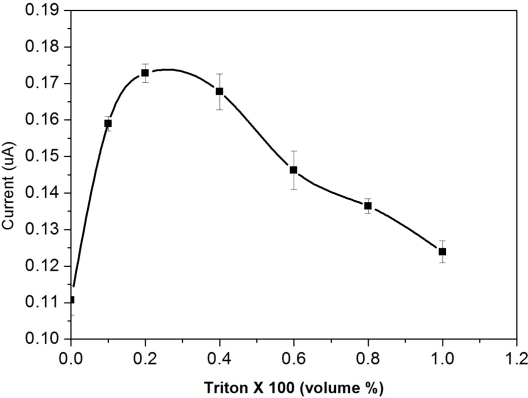
Effect of the surfactant Triton X-100 quantity contained of the disposable biosensor in 0.1 M phosphate buffer (30 μM DG) at potential of +0.5 V, *versus* Ag/AgCl.

The enzymes lipase (22U mL^−1^) and glycerol kinase (1U mL^−1^) were incubated for 1 h prior to adding into the 300 μL test medium. A test medium of 0.1 M phosphate buffer solution with the optimum pH value of 8.3 was used throughout this testing. DG concentration fixed at a level of 30 μM was used, and in each test, 6 μL of the test solution was pipetted onto the surface of the biosensor. The Triton X-100 concentration was varied over the range of 0 to 1% by volume. The oxidation current was then measured at +0.5 V *versus* the Ag/AgCl reference electrode. Figure 5 shows the experimental results on the effect of the quantity of surfactant to the performance of the biosensor prototype.

It appeared clearly that a surfactant concentration of 0.25∼0.28% by volume is the optimal surfactant concentration range to be used in the DG detection. Thus, a surfactant concentration of 0.25% by volume was chosen for DG detection based on the results of this study.

### 3.5. Examination of Optimal Concentration of Lipase, GK and GPD

There are three enzymes used in this biosensor development as shown in equations 1–3. Our objective in this research is to develop a single use, disposable biosensor for DG detection using small sample volumes of blood or other biological fluids. In this study, it was desired to ensure that sufficient quantity of each enzyme will be available to complete the reactions described in equations 1–3. On the other hand, we desire use the minimum quantity of each enzyme needed in order to minimize the cost of producing the biosensors. Enzyme activity can be affected by the presence of other bio-molecules in the test solution, temperature, and pH value of the test medium. Thus, experimental evaluation of the required quantity of each enzyme used in the test medium would be necessary. In a typical experimental evaluation, bovine serum was used as the test medium with the total volume of the serum being 300 μL.

**Figure 6.**
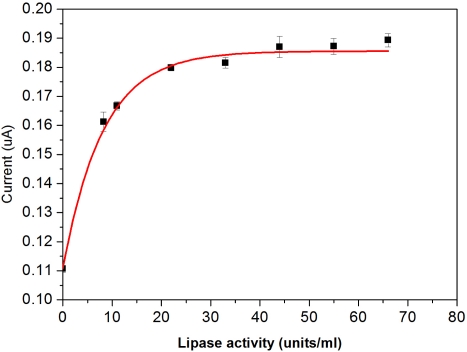
Effect of lipase activity on the hydrolysis of DG in bovine serum.

The enzyme activity of lipase was studied at 37 °C in the presence of 30 μM DG in a 300 μL test medium. The test medium was bovine serum containing ATP (60 μM), GK (1U mL^−1^), BSA, and immobilized GPO (1U mL^−1^) on the working electrode. Without the presence of lipase, a small amount of DG present in the bovine serum before hydrolysis gave a relatively weak response as shown in Figure 6.

The response current increases with an increase of lipase addition, leveling off at about 22U mL^−1^. Hence, for further studies 22U mL^−1^ was used for completion of the hydrolysis of the DG in 1 h.

**Figure 7.**
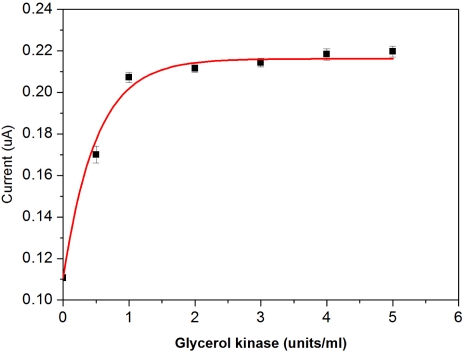
Optimization of glycerol kinase in bovine serum.

The optimum activities of both the soluble glycerol kinase and the immobilized glycerol 3-phosphate oxidase on the working electrode were experimentally assessed similarly. As anticipated, the response current increases with an increase in glycerol kinase addition, as shown in Figure 7.

**Figure 8.**
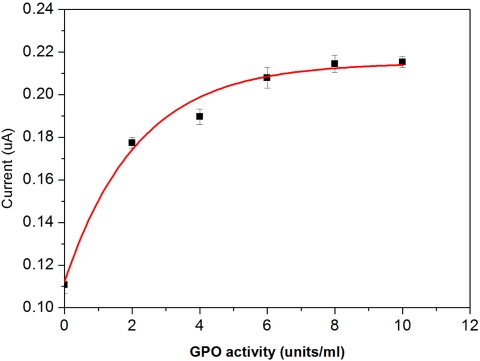
Effect of immobilized glyercol 3-phosphate oxidase on the working electrode in bovine serum.

Based on the results, the quantity of 1U mL^−1^ glycerol kinase was chosen as the optimal concentration level used in this DG biosensor application. The response current also increases with an increase of glycerol 3-phosphate oxidase addition. Based on the results shown in Figure 8, an optimal activity of 6U mL^−1^ of glycerol 3-phosphate oxidase was chosen.

Briefly stated, the study on each enzyme activity in bovine serum as described in this section is essential to determine the needed quantity of each enzyme in the development of a practical single-use, disposable DG biosensor.

### 3.6. Electrochemical Characterization of the Single-Use Disposable Biosensor

In practical applications, this single-use disposable biosensor will be used in a test medium of serum or other physiological fluids. Therefore, this potentially single-use disposable biosensor was evaluated in phosphate buffer and 1:1 bovine serum-buffer. Our experimental results showed that the linear relationship between current and DG level in the bovine serum was limited to 30 μM. At a DG concentration of 40 μM or higher, in bovine serum, the reproducibility of the sensor performance suffered. However, for practical applications, a DG biosensor that could measure the DG level of up to 30 μM would be sufficient for many clinical and point-of-care applications. By diluting the testing sample with known volumes of PBS the detection of DG could be extending to over 50 μM. To illustrate this, 300 μL of phosphate buffer and 1:1 bovine serum-buffer, lipase (22U mL^−1^), ATP (100 μM), GK (1U mL^−1^) and DG were added into a test tube and shaken to obtain a homogeneous mixture and then incubated at 37 °C for 1 h. As mentioned, a period of incubation time was needed because of the kinetics of the enzymatic reactions, especially at higher DG concentrations. The performance of this single-use, disposable biosensor was investigated using amperometric measurements in the testing solutions, phosphate buffer and 1:1 bovine serum-buffer solution with an applied potential at +0.5 V *versus* Ag/AgCl reference electrode. Biosensors without DG were also tested under the same experimental condition for comparison purpose.

**Figure 9.**
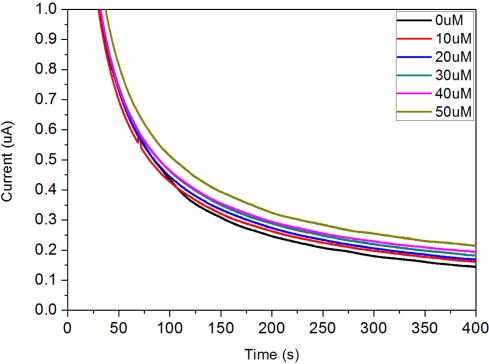
Amperometric response of the disposable biosensor to different DG concentrations. Test medium was 0.1 M pH 8.3 phosphate buffer. Applied potential was +0.5 V *versus* Ag/AgCl.

The performance of this DG biosensor prototype was evaluated using the optimal pH, surfactant concentration and enzyme loading determined previously determined. The amperometric response of this single-use disposable biosensor for various concentrations of DG at +0.5 V *versus* the Ag/AgCl reference electrode in the presence of 0.1 M phosphate buffer pH 8.3 is shown in Figure 9.

**Figure 10.**
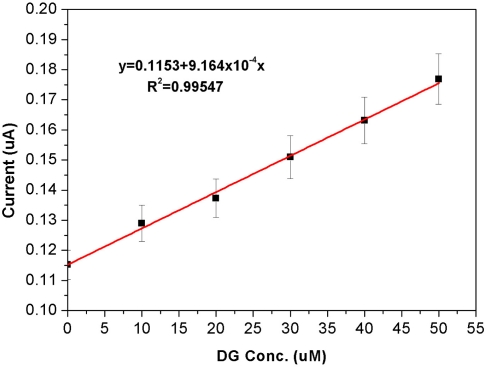
Calibration curve obtained at 300 s for the DG measurement in the 0.1 M pH 8.3 phosphate buffer. Good linear relationship is between response current and DG concentration is observed with a general equation of Y = 9.164 × 10^−^^4^X + 0.1153 and R^2^ = 0.995.

The results show a clear trend in response current *versus* DG concentration. Thus successful detection of DG using the disposable biosensor was accomplished. A calibration curve obtained by plotting the response current at 300 s *versus* DG concentration is shown in Figure 10.

**Figure 11.**
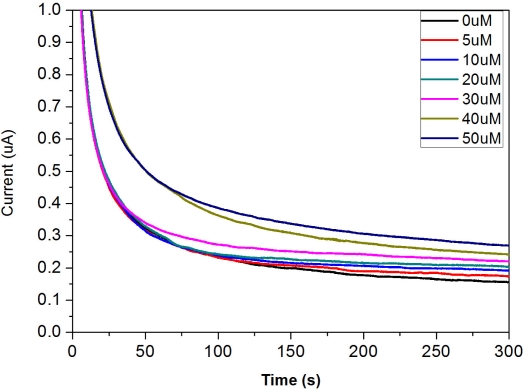
Amperometric response of the disposable biosensor to different DG concentrations in 1:1 bovine serum-buffer medium.

The testing range of DG concentration from 0 to 50 μM in bovine serum was also examined. However, a well fit trend of response current was only suitable up to a concentration of 30 μM (data not shown). To extend the testing range the bovine serum was diluted and the trend of response current was studied. In this study a 1:1 dilution of bovine serum-buffer was chosen. BSA was also added into the test medium because the bovine serum has many miscellaneous proteins. The dependence of the amperometric response for this single use, disposable biosensor on the different concentrations of DG is shown in Figure 11.

**Figure 12.**
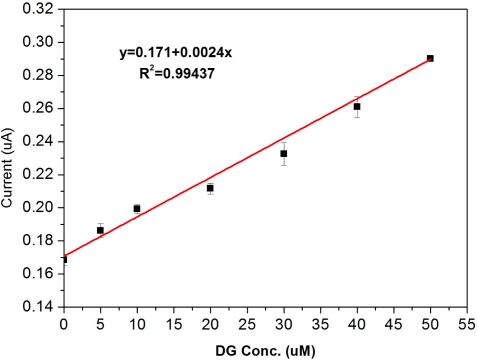
Calibration curve obtained at 240 s for the DG measurement in 1:1 bovine serum-buffer medium.

The results show a clear trend of different DG concentrations and the dependence of response current on DG concentration. The response current in 1:1 bovine serum-buffer was higher than the response current in phosphate buffer. In general, the response current in serum was lower than the response current in buffer because of its low diffusion rate; however, it was case by case. In our study, the reason for higher response current in 1:1 bovine serum-buffer may be due to the presence of various ions in the serum that enhanced the conductivity of the test medium. Figure 12 shows calibration plot obtained at 240 s based on the amperometric results shown in Figure 11.

**Figure 13.**
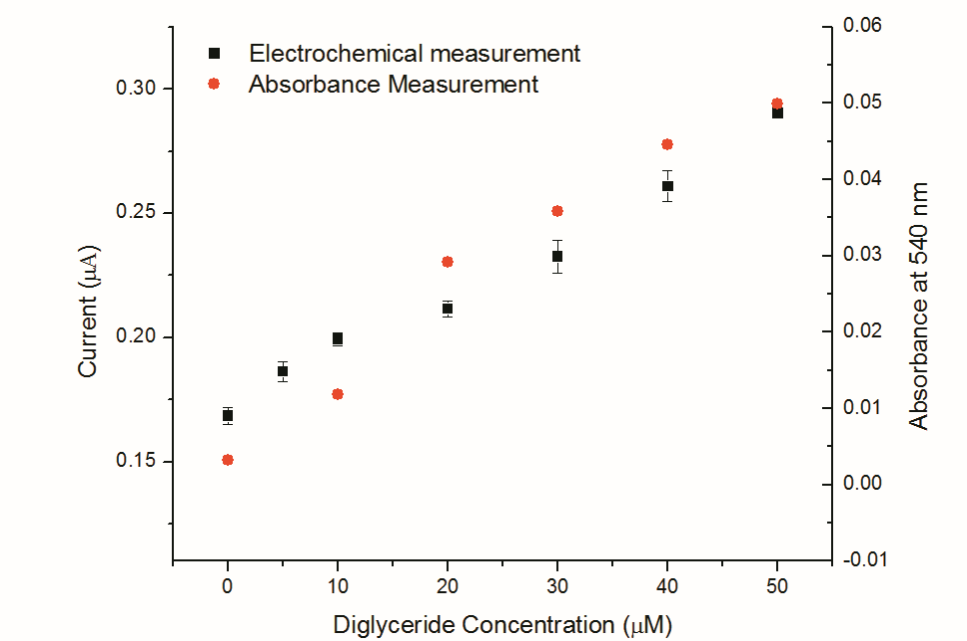
Comparison of 1:1: bovine serum-buffer samples containing DG measure by sepctrophotometry and the proposed single-use biosensor.

A linear relationship was observed for DG concentrations ranging from 0 to 25 μM (because of the diluted serum). The result of linear fit was Y = 0.0024X + 0.171 and the R^2^ = 0.994. These results indicated that the single use, disposable biosensor would be a good candidate for DG detection in bovine serum. Finally, the current response of the single-use biosensor was compared to readings taken from a spectrophotometer. As this measurement was often considered the “gold standard” for the enzymatic based assays, efforts were conducted to understand the two measurement techniques compared. The comparison shows that good linear performance is observed in both measurement techniques. Based on this confirmation, it is reasonably to suggest that the single-use, disposable DG biosensor would be suitable to be used as a simple and cost effective means to measure DG in a serum sample using the established experimental protocol described. Spectrophotometric studies were conducted using an assay purchased from Cayman Chemical (Ann Arbor, MI). Samples were prepared in a 96-well tray and absorbance was measured at 540 nm using a standard plate reader. A comparison of the two measurement techniques is shown in Figure 13.

The comparison shows that good linear performance is observed in both measurement techniques. Based on these results, one can reasonably suggest that this single-use biosensor would be suitable as a potential replacement to spectrophotometric measurement of DG in serum.

## 4. Conclusions

A single use, disposable biosensor for the enzymatic determination of DG was developed. Experimental results show that lipase (22U mL^−1^), glycerol kinase (1U mL^−1^) and glycerol 3-phosphate oxidase (6U mL^−1^) were the optimal enzyme activity levels for this biosensor prototype. In order to improve the solubility of DG, 0.28% (v/v) of surfactant Triton X-100 was added into the test medium. An applied potential of +0.5 V *versus* Ag/AgCl was chosen, based on cyclic voltammetric studies, as the working potential of this DG detection system. Within the testing range of 0–50 μM of DG, a good linear relationship between current response and DG level was observed in both PBS and bovine serum diluted with PBS. Studies also showed the sensor to have good sensor to sensor reproducibility. Future research efforts related to this sensor will include the impact of interfering species and the role of storage conditions on sensor performance.
